# Expression pattern of genome-scale long noncoding RNA following acute myocardial infarction in Chinese Uyghur patients

**DOI:** 10.18632/oncotarget.16355

**Published:** 2017-03-18

**Authors:** Hui Zhai, Xiao-Mei Li, Fen Liu, Bang-Dang Chen, Hong Zheng, Xue-Mei Wang, Wu Liao, Qing-Jie Chen, Yi-Tong Ma, Yi-Ning Yang

**Affiliations:** ^1^ Department of Cardiology, The First Affiliated Hospital of Xinjiang Medical University, Urumqi, China; ^2^ Xinjiang Key Laboratory of Cardiovascular Disease Research, Urumqi, China; ^3^ Anesthesia Department, The First Affiliated Hospital of Xinjiang Medical University, Urumqi, China

**Keywords:** long non-coding RNA, acute myocardial infarction, microarray

## Abstract

In this study, we examined the long noncoding RNA (lncRNA) expression pattern in Uyghur patients (a minority of China) with acute myocardial infarction (AMI) on a genome-wide scale. Total RNAs were extracted from the peripheral blood of 55 Uyghur AMI patients and 55 healthy volunteers. The expression levels of genome-wide scale lncRNAs and mRNAs were determined by microarray in 10 samples (5 AMI and 5 controls). qRT-PCR was used to validate lncRNA expression levels in 100 samples (50 AMI and 50 controls). Data analyses were performed using R and Bioconductor. A total of 3624 up- and 1637 down-regulated lncRNAs were identified to be significantly and differentially expressed between these two groups. The annotation result of their co-expressed mRNAs showed that the most significantly related category of GO analysis was regulation of biological processes, and the most significantly related pathway was apoptosis and its corresponding p53. The microarray identified ENST00000416860.2, ENST00000421157.1 and TCONS_00025701 lncRNAs were confirmed by qRT-PCR. Our study indicated that clusters of lncRNAs were significantly and differentially expressed in the peripheral blood of AMI patients when compared with healthy controls within the Uyghur population. These newly identified lncRNAs may have a potential role in the development of AMI.

## INTRODUCTION

Acute myocardial infarction (AMI) is one of the most serious cardiovascular diseases worldwide, as it is associated with high rates of morbidity and mortality. Multiple conventional risk factors and serum atherosclerotic biomarkers have been established for coronary artery disease (CAD) [1]. Advances in genomics and proteomics, especially gene-expression profiling using microarray have promoted the development of many novel molecular biomarkers with potential clinical values for AMI [2, 3]. Recently, long non-coding RNAs (lncRNAs) have attracted extensive interest in the field of cardiovascular diseases [4].

LncRNAs are a group of RNA transcripts with > 200 nucleotides [5]. Although they lack protein coding capability, lncRNAs play an important role in many biological processes, including RNA-RNA interactions and epigenetic and post-transcription regulation [6]. It has been reported that lncRNAs play a role in regulating cardiac development and in the pathogenesis of heart failure [7–9]. For example, a lncRNA named cardiac hypertrophy related factor (CHRF) has been shown to regulate cardiac enlargement [10]. It is believed that lncRNAs also play a role in cardiovascular ageing [11]. Recently, Ounzain S et al [12] reported that expression levels of lncRNAs were altered in the cardiac tissue after AMI, which extended the pathophyiological role of lncRNA into AMI. The Uyghur is a Muslim minority in China, Uyghur population is count for 48.5% population in Xinjiang, the largest northwest province in China. The Uyghur has a unique lifestyle with a high incidence of CAD [13]. While expression of genome-scale lncRNAs and their potential biological functions in Uyghur AMI patients remain obscure.

Therefore, using microarray analysis, we investigated the expression of lncRNAs in peripheral blood cells of 5 Uyghur AMI patients and made comparison with matched healthy control subjects. The results were further validated by qRT-PCR in 50 Uyghur AMI patients and 50 Uyghur healthy controls. The differentially expressed lncRNAs were identified in the two groups and then integrated with mRNA expression data to predict the possible function of these lncRNAs.

## RESULTS

### Patients’ enrollment and lncRNA categorization

This study recruited 55 AMI patients and 55 matched healthy controls. The clinical characteristics of study participants are shown in Table 1. Peripheral blood samples were collected and total RNA was extraction. Expression profile of lncRNA and mRNA from 5 AMI patients and 5 matched healthy controls were detected using microarray, and the rest study participants were used for validation. In this study, we determined the expression of a total of 41,000 lncRNA probes. According to the categorization of lncRNAs by Clark et al in the GENCODE gene annotation [14], we classified all tested lncRNAs into 6 groups: sense, antisense, intronic, intergenic, bidirectional and unknown. The classification criteria were based on the lncRNA location with respect to protein-coding genes.

**Table 1:** Clinical characteristics of study participants

	Healthy Control *n* = 55	AMI patient *n* = 55
Age, years	63.8 ± 9.3	52.6 ± 10.9*
sex, male/female	38/17	29/26
Heart rate, beats per minute	73.9 ± 9.0	76.6 ± 10.4
BMI (kg/m2)	25.8 ± 3.2	25.6 ± 3.6
Systolic blood pressure, mmHg	129 ± 17	126 ± 14
Diastolic blood pressure, mmHg	76 ± 9	80 ± 12
Diabetes mellitus, %	13/42	9/46
Smoking,%	28/27	23/32
Drinking, %	20/35	11/44*
Glucose, mmol/L	4.9 ± 0.6	7.5 ± 1.3*
TC, mmol/L	1.8 ± 0.3	4.2 ± 1.3*
TG, mmol/L	1.9 ± 1.2	2.5 ± 1.2*
HDL, mmol/L	0.8 ± 0.1	1.0 ± 0.1*
LDL, mmol/L	3.0 ± 0.2	3.5 ± 0.6*

Data are mean value ± standard deviation. BMI, body mass index; TC, total cholesterol; TG, triglycerides; HDL, high density lipoprotein; LDL, low density lipoprotein. *P < 0.05 compared with healthy control.

### Identification of distinct lncRNA expressions in AMI patients

**Figure 1:**
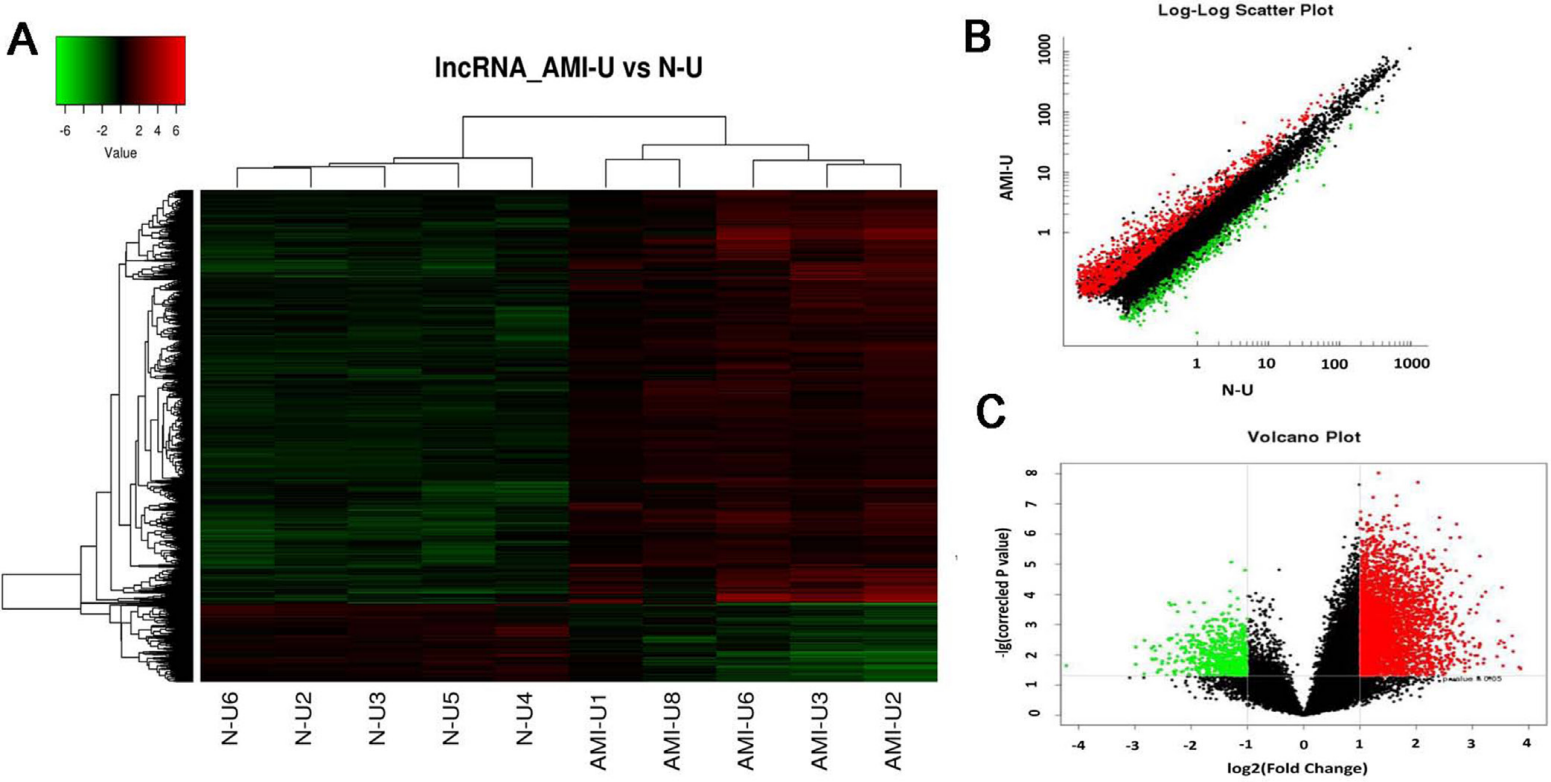
Differential expression of lncRNAs in patients with acute myocardial infarction (AMI) and control individuals in Uyghur of Chinese Hierarchical clustering analysis of 5261 lncRNAs that were differentially expressed in the two groups. (**A**) Expression values are represented in red and green, indicating expression above and below the median expression value in AMI patients (AMI-U1, AMI-U2, AMI-U3, AMI-U6, AMI-U8) or control individuals (N-U2, N-U3, N-U4, N-U5, N-U6), respectively. (**B**) Scatter plot of differential lncRNA expression. X-axis: N-U, Y-axis: AMI-U. (**C**) Volcano plot of differential lncRNA expression. X-axis: log2 fold change; Y-axis: −1 × log10 (corrected *p-value*) for each probes.

We first analyzed the locus-by-locus lncRNA probes from the peripheral blood of AMI patients (*n* = 5) and healthy controls (*n* = 5). By the criteria of a corrected *P* value < 0.05 and an absolute fold change (FC) > 2.0, we identified 3624 up-regulated and 1637 down-regulated lncRNAs that were significantly and differentially expressed between the two groups. The top 25 of distinctively expressed lncRNAs according to the FC values in Uyghur AMI patients are presented in Table 2. As hierarchical clustering represents one of the simplest and most widely used clustering techniques for analysis of lncRNA and gene expression data which can then enable the generation of hypotheses about the relationships among samples [15], we adopted this technique in our experiment as well. The results of hierarchical clustering in these 10 samples (5 AMI patients and 5 healthy controls) showed distinguishable lncRNAs expression profiles (Figure 1A). The scatter and volcano plots are presented as visualizations used to assess lncRNA expression variation between the two groups (Figure 1B–1C). Every dot represents a lncRNA. The red dots represent the up-regulated lncRNAs (FC value > 2, *P* < 0.05), the green dots represent the down-regulated lncRNAs (FC value < –2, *P* < 0.05), the black dots represent the rest of lncRNAs (–2 < FC value < 2, *P* > 0.05).

**Table 2:** The top 25 of differentially expressed lncRNAs according to the fold change (FC) values in AMI patients of uyghur chinese compared with that in healthy controls

lncRNA ID	Probe name	FC value	Regulation	class	database
**ENST00000541341.1**	p33400	18.66	down	Antisense	ENSEMBL
**TCONS_00024652**	p33618	14.48	up	Antisense	HumanLincRNACatalog
**ENST00000424119.1**	p9163	14.21	up	Intergenic	ENSEMBL
**TCONS_00012852**	p23866	13.18	up	Intergenic	HumanLincRNACatalog
**ENST00000365067.1**	p38671_v4	13.04	up	unkown	ENSEMBL
**TCONS_00011823**	p23452	11.93	up	Divergent	HumanLincRNACatalog
**ENST00000363358.1**	p38670_v4	11.76	up	unkown	ENSEMBL
**ENST00000508732.2**	p5368	11.64	up	Intergenic	ENSEMBL
**ENST00000561259.1**	p5097	11.50	up	Intergenic	ENSEMBL
**TCONS_00004897**	p21231	11.30	up	Intergenic	HumanLincRNACatalog
**uc031szk.1**	p44257_v4	11.05	up	unkown	UCSC
**TCONS_00020349**	p18802	11.00	up	Intergenic	HumanLincRNACatalog
**TCONS_00004284**	p20855	10.82	up	Intergenic	HumanLincRNACatalog
**ENST00000565523.1**	p6372	9.41	up	Intergenic	ENSEMBL
**uc002ywy.3**	p26170	9.22	up	Intronic	UCSC
**ENST00000434741.1**	p11186	9.15	up	Intergenic	ENSEMBL
**TCONS_00014223**	p23868	8.77	up	Intergenic	HumanLincRNACatalog
**ENST00000566676.1**	p34425_v4	8.77	up	Intergenic	ENSEMBL
**ENST00000518846.1**	p16012	8.76	up	Intergenic	ENSEMBL
**ENST00000563515.1**	p6219	8.67	up	Intergenic	ENSEMBL
**ENST00000597626.1**	p10862	8.56	up	Antisense	ENSEMBL
**ENST00000424640.2**	p36969_v4	8.50	up	unkown	ENSEMBL
**ENST00000365306.1**	p38669_v4	8.50	up	unkown	ENSEMBL
**ENST00000447911.2**	p5449	8.27	up	Intergenic	ENSEMBL
**TCONS_00004013**	p21307	8.22	up	Intergenic	HumanLincRNACatalog

**Figure 2:**
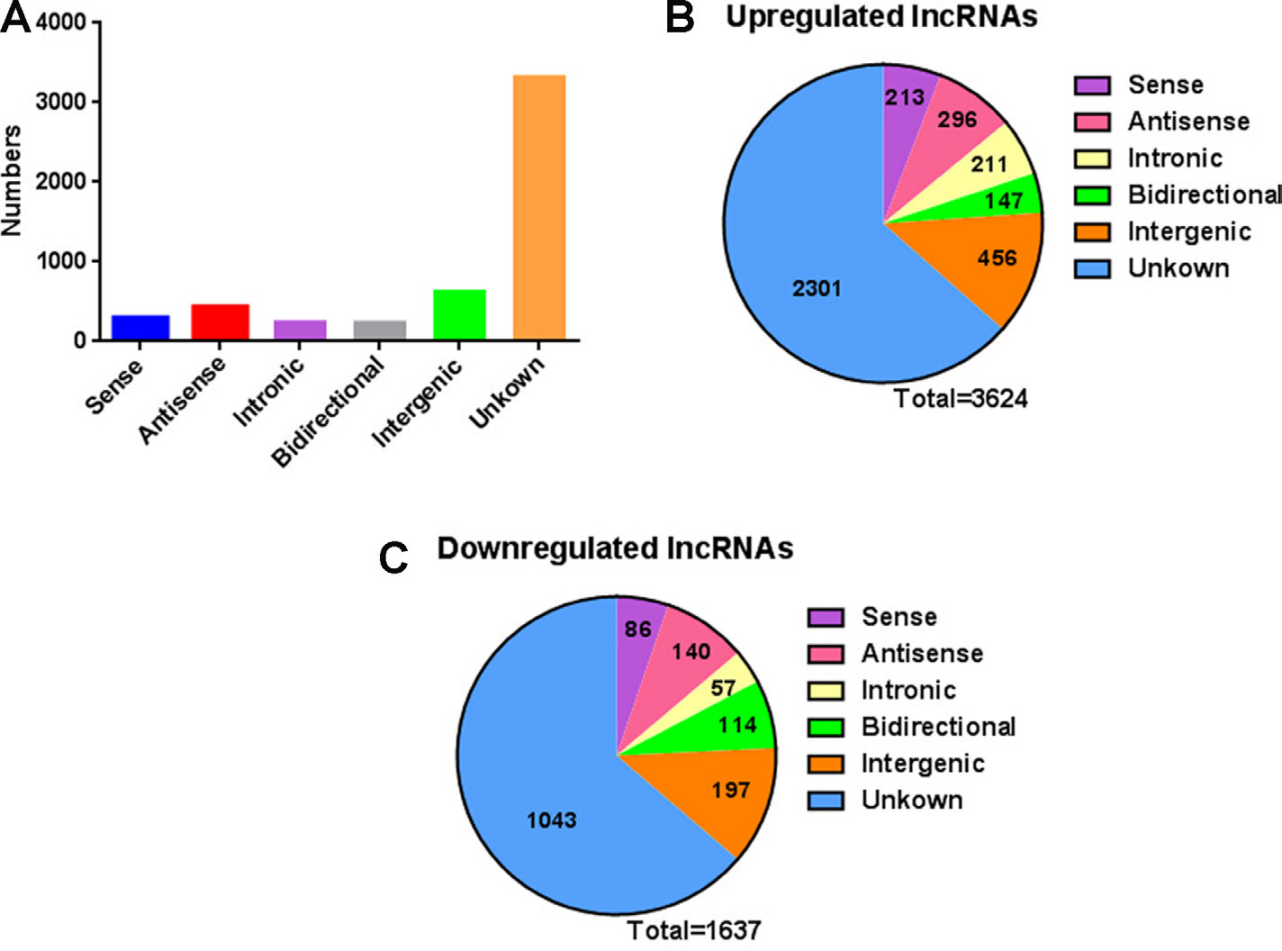
The classification of lncRNAs into six categories (sense, antisense, intronic, intergenic, bidirectional and unknown) according to their relationships with protein-coding genes (**A**) The numbers of identified lncRNAs in different categories. (**B**) Pie charts showing the number of up-regulated lncRNAs in each category. When a criteria of fold change > 2 and *P* < 0.05 was accepted, 3624 lncRNAs were up-regulated. (**C**) Pie charts showing the number of down-regulated lncRNAs in each category. When a criteria of fold change > 2 and *P* < 0.05 was accepted, 1637 lncRNAs were down-regulated.

**Figure 3:**
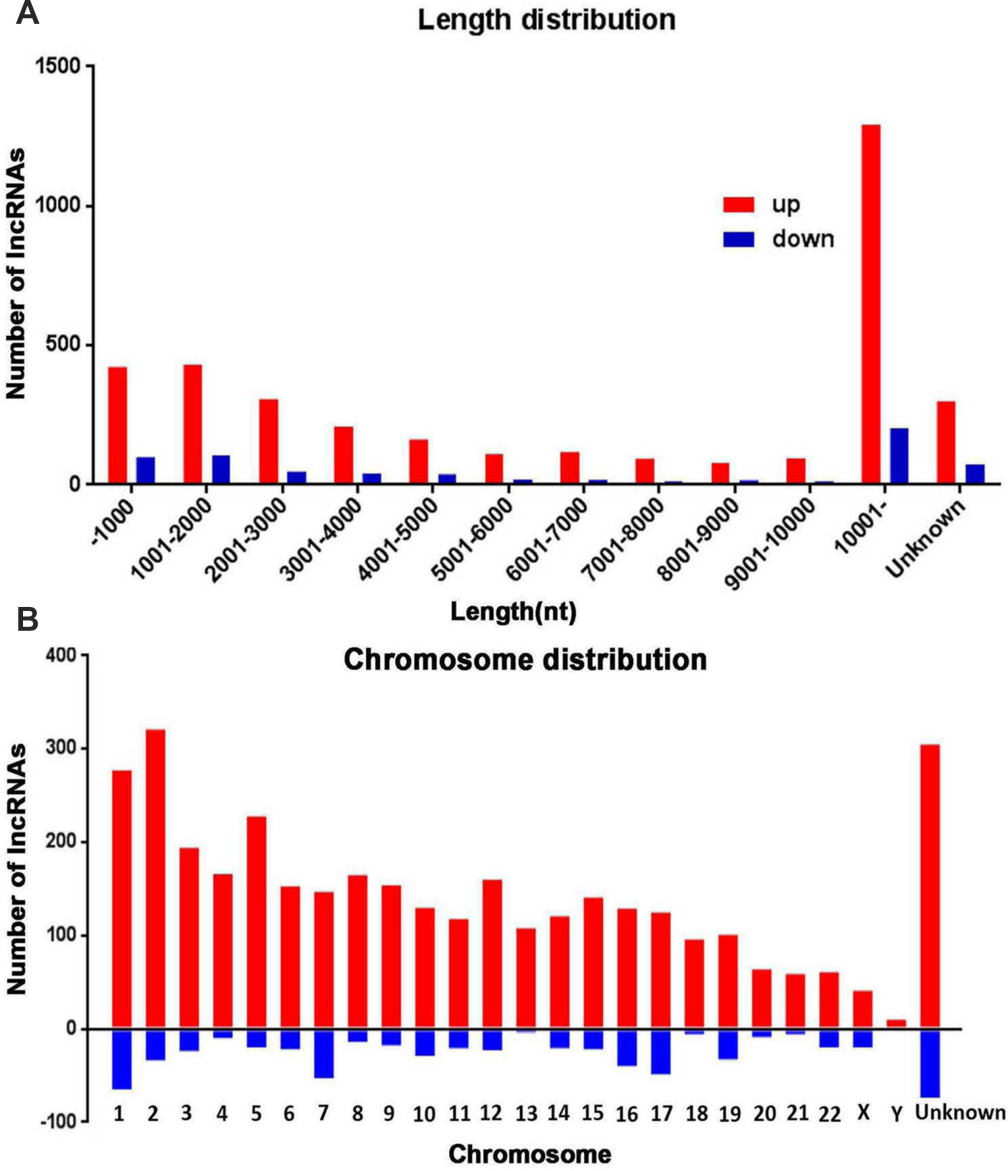
The length and chromosomes distribution of dysregulated lncRNAs (**A**) The length distribution of dysregulated lncRNAs. (**B**) Distribution of up and down regulated lncRNAs location in different chromosomes.

Next, we analyzed distinctive lncRNAs based on their categorizations. Although many lncRNAs were not categorized (62.9%), among these classified lncRNAs, the greater proportions were intergenic (12.3%) and antisense (8.2%) versus that of bidirectional (4.9%), sense (5.6%) and intronic (5.0%) (Figure 2A). This pattern was also presented in Figure 2B–2C with regard to up- or down-regulated lncRNAs. The lengths of dysregulated lncRNAs were showed in Figure 3A. The chromosome distribution showed the number of up- and down-regulated lncRNAs located within each corresponding chromosome (Figure 3B).

### Identification of potentially functional lncRNAs in AMI

**Figure 4:**
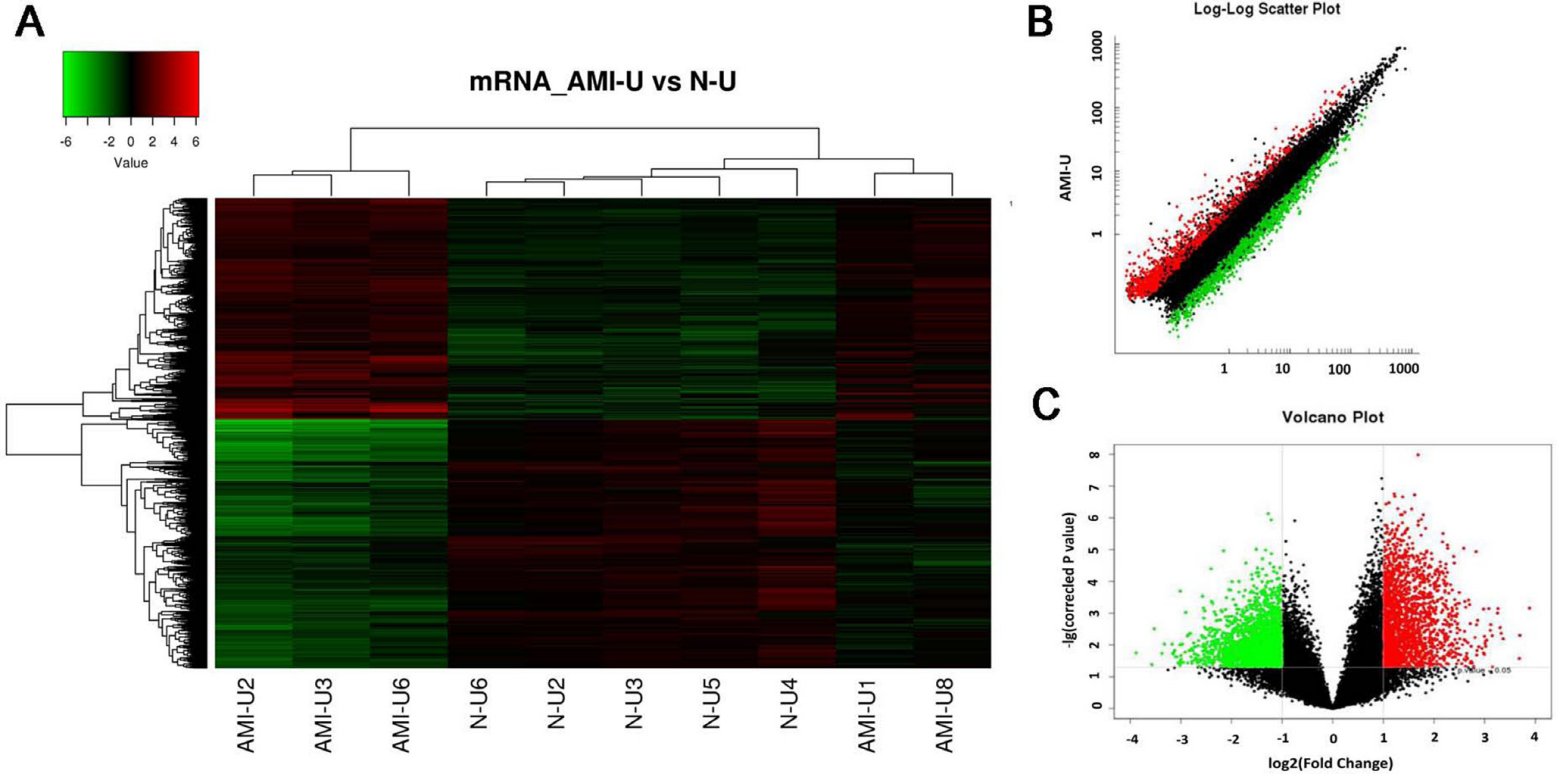
Differential expression of mRNAs related to corresponding lncRNAs in patients with acute myocardial infarction (AMI) and control individuals in Uyghur ethnicity Hierarchical clustering analysis of 3896 mRNAs that were differentially expressed in the two groups. (**A**) Expression values are represented in red and green, indicating expression above and below the median expression value in AMI patients (AMI-U1, AMI-U2, AMI-U3, AMI-U6, AMI-U8) or control individuals (N-U2, N-U3, N-U4, N-U5, N-U6), respectively. (**B**) Scatter plot of differential mRNA expression. X-axis: N-U, Y-axis: AMI-U. (**C**) Volcano plot of differential mRNA expression. X-axis: log2 fold change; Y-axis: −1 × log10 (corrected *P*-value) for each probes.

The function of most identified 5261 differentially expressed lncRNA probes remains unknown. In this experiment, we predicted their potential function via the annotation of their co-expressed mRNAs. First, expression profile of the genome-wide mRNA in these 5 AMI patients and 5 healthy controls were examined by microarray. Again, the criteria of a corrected *P* value < 0.05 and an absolute FC > 2 were used to identify significantly and differentially expressed mRNAs. Among the 34000 detected mRNA probes, a total of 3896 were found to be significantly and differentially expressed (Figure 4A). Of these mRNAs, 1833 were up-regulated and 2063 were down-regulated. The top 25 of distinctively expressed mRNAs according to the FC values in Uyghur AMI patients are presented in Table 3. The scatter and volcano plots generated from these differentially expressed mRNAs are clearly segregated between the AMI and healthy control clusters (Figure 4B–4C). Every dot represents a mRNA. The red dots indicate the up-regulated mRNAs (FC value > 2, *P* < 0.05), the green dots indicate the down-regulated mRNAs (FC value < –2, *P* < 0.05), the black dots indicate the rest of the mRNAs (–2 < FC value < 2, *P* > 0.05). This result suggests that these mRNAs were substantially different between the AMI patients and healthy controls.

**Table 3:** The top 25 of distinctively expressed mRNAs according to the fold change (FC) values in AMI patients of Uyghur Chinese compared with that in healthy controls

Probe name	*P* value	FC value	Regulation	Gene Symbol	Ensembl ID
**A_23_P130689**	0.017	14.76	down	ELOF1	ENST00000590700
**A_21_P0009030**	0.027	12.87	up	C16orf97	ENST00000562818
**A_23_P407074**	0.042	11.90	down	DNM2	ENST00000408974
**A_24_P14367**	0.003	11.50	down	PTBP1	ENST00000394601
**A_24_P134235**	0.0183	10.37	down	KHSRP	ENST00000619396
**A_24_P63019**	0.006	10.30	up	IL1R2	ENST00000457817
**A_24_P276490**	0.010	10.01	down	LYPLA2	ENST00000420982
**A_33_P3319967**	0.003	9.89	up	ARG1	ENST00000356962
**A_23_P160902**	0.001	9.61	up	FCAMR	ENST00000324863
**A_33_P3314500**	0.001	9.54	up	MUC6	ENST00000619759
**A_21_P0000664**	0.009	9.02	down	CD4	ENST00000544344
**A_33_P3589217**	0.019	8.91	down	SLC25A6	ENST00000484026
**A_23_P118289**	0.018	8.86	down	BCL7C	ENST00000215115
**A_23_P125771**	0.015	8.72	down	HCFC1	ENST00000310441
**A_24_P400604**	0.003	8.65	up	RBMY1B	ENST00000623132
**A_23_P404678**	0.024	8.61	down	RAB3D	ENST00000222120
**A_33_P3262969**	0.001	8.48	up	COL4A6	ENST00000461897
**A_23_P56213**	0.024	8.46	down	GRAMD1A	ENST00000600231
**A_24_P224727**	0.037	8.30	down	CEBPA	ENST00000498907
**A_21_P0003860**	0.007	8.24	up	LOC101928223	ENST00000504509
**A_33_P3331952**	0.011	8.11	up	OR2M3	ENST00000456743
**A_23_P29851**	0.010	8.10	down	LRPAP1	ENST00000515119
**A_33_P3245575**	0.043	8.09	down	COLGALT1	ENST00000597075
**A_23_P152024**	0.033	8.08	down	CSK	ENST00000564216
**A_33_P3269539**	0.000	8.07	down	COL6A2	ENST00000397763

**Figure 5:**
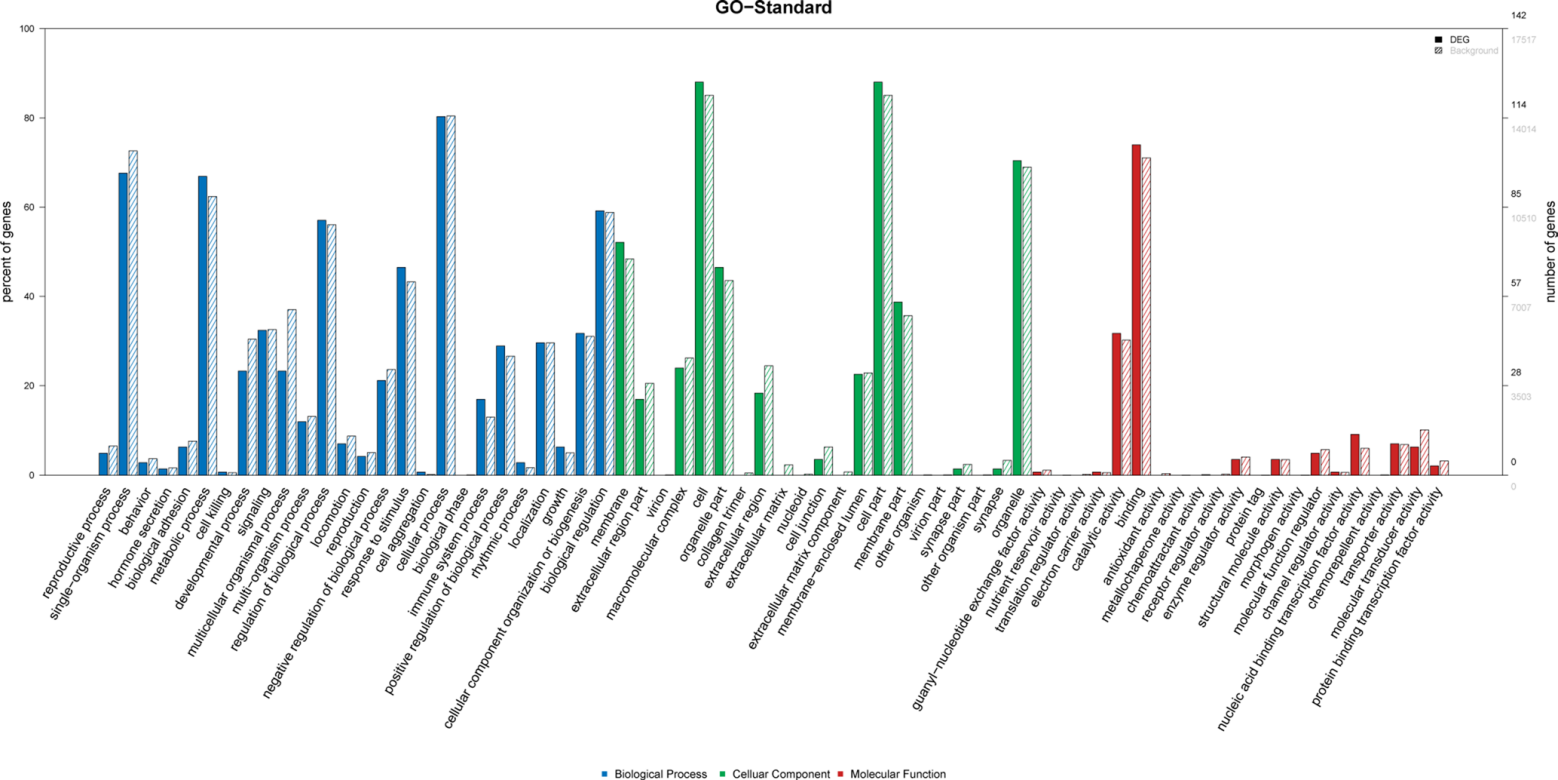
GO analysis of differentially expressed lncRNAs which covers three domains: biological process, cellular component and molecular function X-axis: GO terms of biological process, cellular component and molecular function. The blue column indicates biological process, the green column indicates cellular component and the red column indicates molecular function. Y-axis on the left: percentage of genes (lncRNAs); Y-axis on the right: numbers of genes (lncRNAs).

Among the Gene Ontology (GO) terms enriched up-regulated lncRNAs, the top 10 terms associated with biological processes in AMI patients included: (1) reproductive processes, (2) single-organism processes, (3) behavior, (4) hormone secretion, (5) biological adhesion, (6) metabolic processes, (7) cell killing, (8) developmental processes, (9) signaling and (10) multicellular organismal processes. The top 10 GO terms related to cellular components in AMI patients included: (1) membrane, (2) extracellular region, (3) virion, (4) macromolecular complex, (5) cell, (6) organelle, (7) collagen trimer, (8) extracellular region, (9) extracellular matrix and (10) nucleoid. The top 10 GO terms related to molecular function activity of AMI patients were the followings: (1) guanyl-nucleotide exchange factor, (2) nutrient reservoir, (3) translation regulator, (4) electron carrier, (5) catalytic, (6) binding, (7) antioxidant, (8) metallochaperone, (9) chemoattractant and (10) receptor regulator (Figure 5).

**Figure 6:**
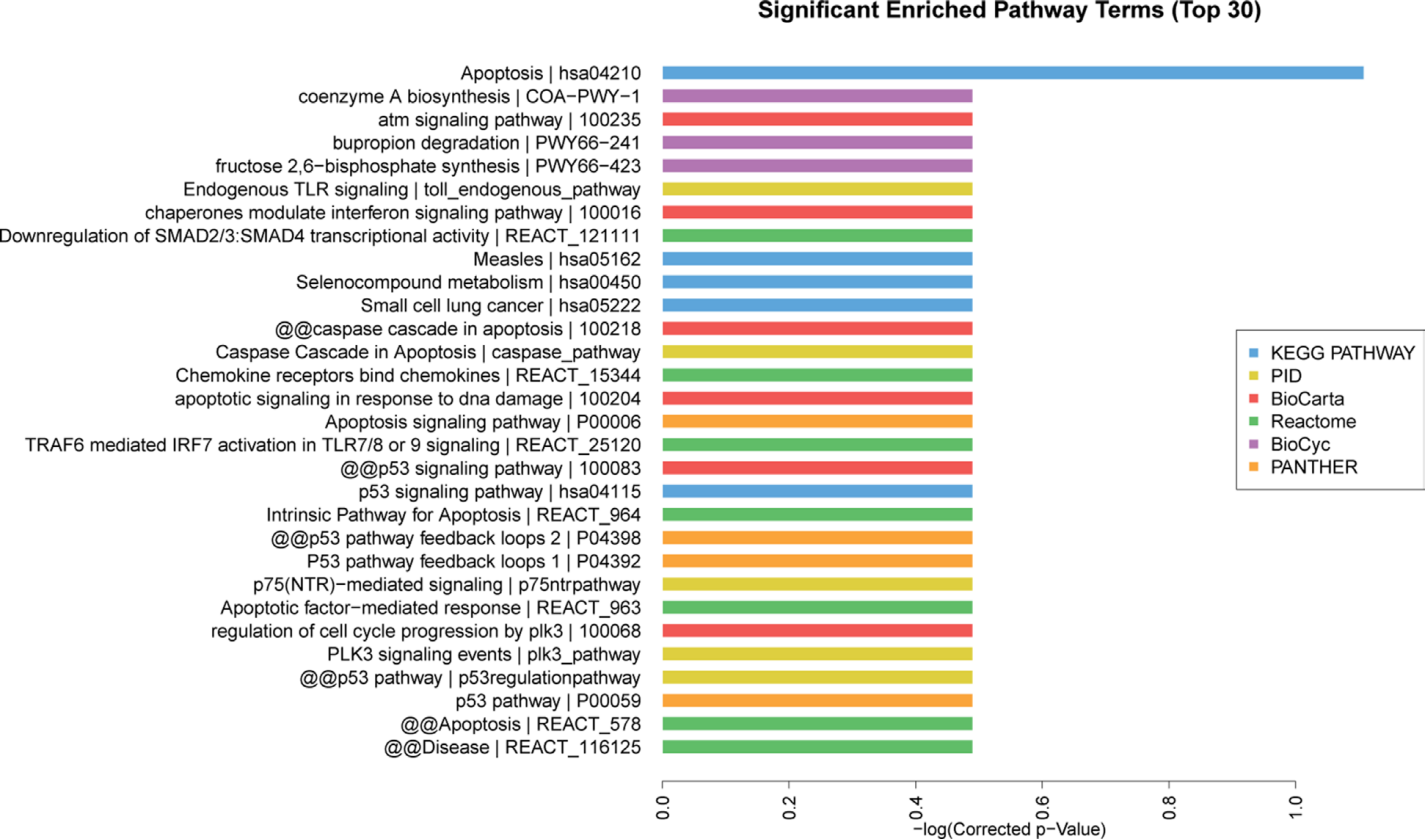
Pathway analysis of differentially expressed lncRNAs The top 30 significantly enriched pathways were calculated and plotted as the −1 × log10 (*P*-value). Pathway analysis is a functional analysis mapping genes to KEGG pathway and other pathway databases. Different colors represent different databases. The lower the *P*-value, the more significant the pathway.

**Figure 7:**
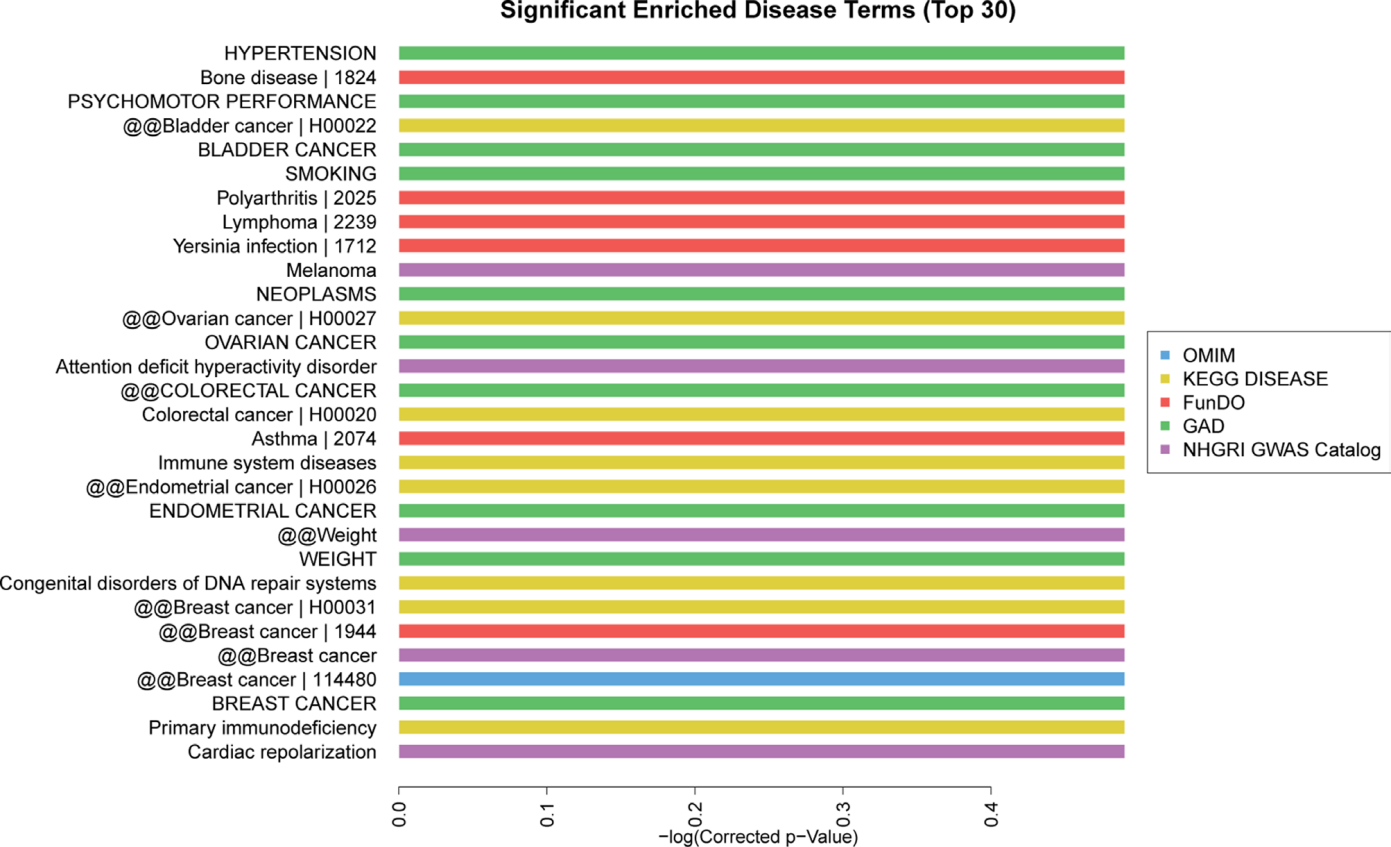
Disease analysis of differentially expressed lncRNAs The top 30 significantly enriched disease terms were calculated and plotted as the −1 × log10 (*P*-value). The disease terms derived from five database: OMIM, KEGG disease, FunDO, GAD, NHGRI GWAS Catalog, which were indicated in blue, yellow, red, green, purple, respectively. The bar graph shows the top 30 enrichment score [-log10 (*P*-value)] value of the significantly enriched diseases.

Based on the Kyoto Encyclopedia of Genes and Genomes (KEGG) pathway analysis, the top 30 significant enriched pathway terms were acquired from 6 databases (KEGG PATHWAY, PID, BioCarta, Reactome, BioCyc and PANTHER). The most enriched pathways corresponding to the dysregulation of lncRNAs as related to AMI were apoptosis (8/30) and p53 pathway regulation (6/30) (Figure 6). The top 10 significant enriched disease terms include: (1) hypertension, (2) cardiac repolarization, (3) primary immunodeficiency, (4) breast cancer, (5) congenital disorders of DNA repair systems, (6) weight, (7) endometrial cancer, (8) immune system diseases, (9) asthma and (10) colorectal cancer (Figure 7). The top 30 terms involving these GO and KEGG pathways are presented in Figures 5, 6, 7.

**Figure 8:**
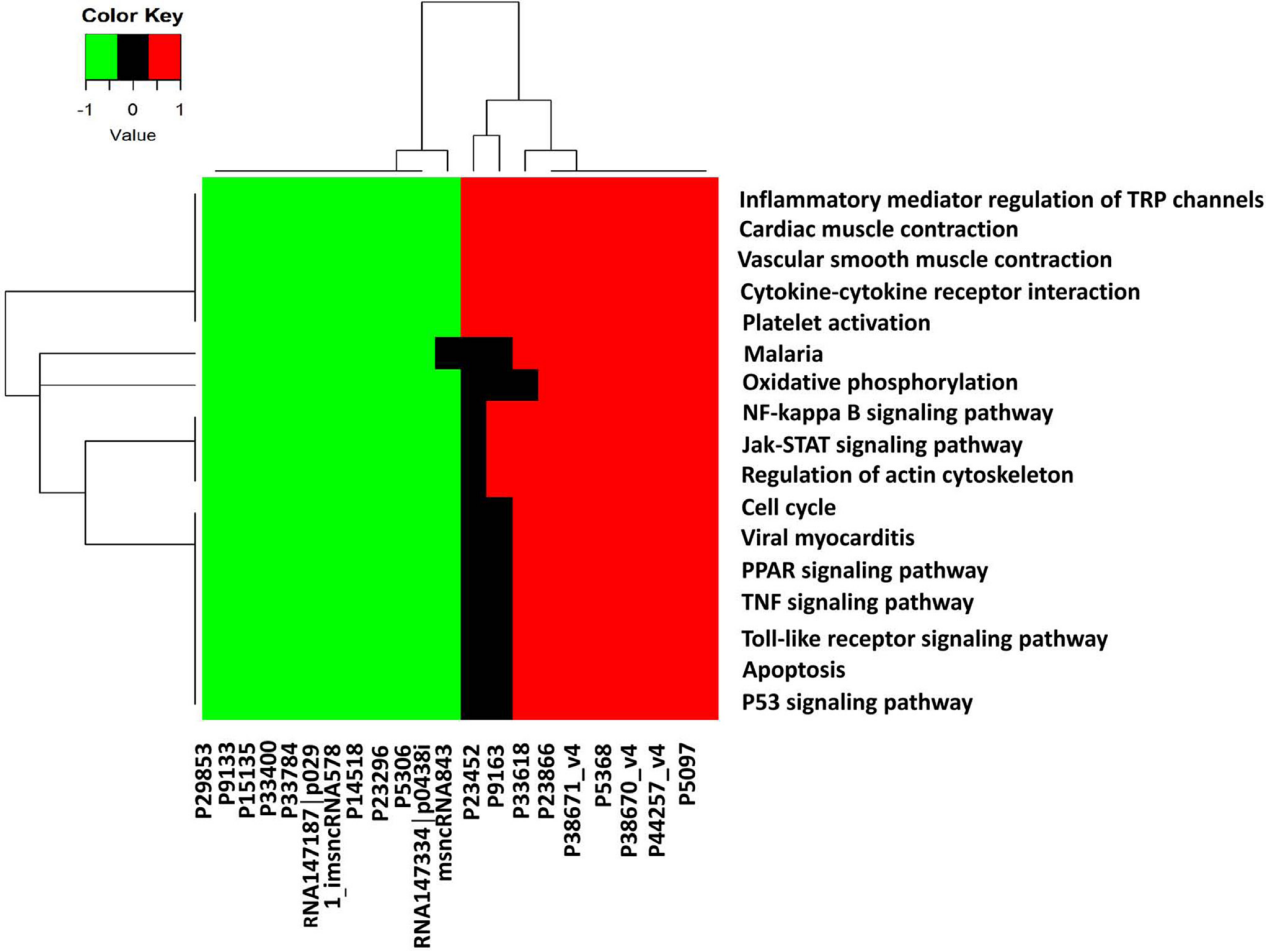
Kyoto encyclopedia of genes and genomes annotation for neighbor gene functions of predicated lncRNAs Hierarchical clustering analysis of 20 lncRNAs that were differentially expressed in the KEGG pathway terms. X-axis on the right (red): 10 higher expressed lncRNAs, X-axis on the left (green): 10 lower expressed lncRNAs. X-axis on the middle (black): no significantly expressed lncRNAs.

We then performed microarray analysis to investigate the corresponding gene changes of aberrantly expressed lncRNA for functional linking study. We found that the top 10 up- and 10 down-regulation lncRNAs were related to (1) inflammatory mediator regulation of Transient Receptor Potential (TRP) channels, (2) cardiac muscle contraction, (3) vascular smooth muscle contraction, (4) cytokine-cytokine receptor interaction, (5) platelet activation, (6) Malaria, (7) oxidative phosphorylation, (8) NF-kappa B signaling pathway, (9) JAK-STAT signaling pathway, (10) regulation of actin cytoskeleton, (11) cell cycle, (12) viral myocarditis, (13) TNF signaling pathway, (14) PPAR signaling pathway, (15) Toll-like receptor signaling pathway, (16) apoptosis, and (17) p53 signaling pathway (Figure 8).

### LncRNA-mRNA network analysis

**Figure 9:**
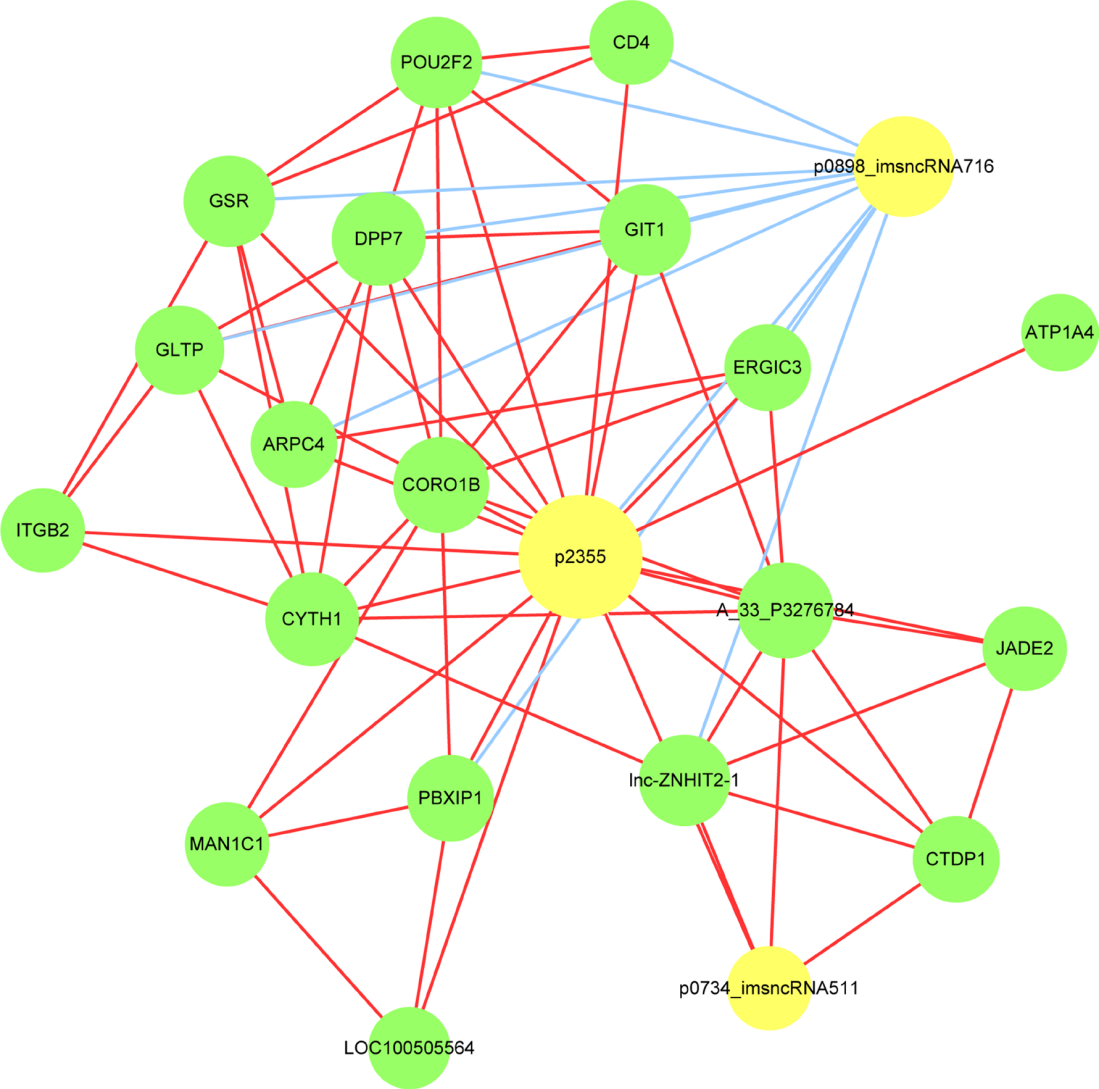
LncRNA-mRNA-network was constructed based on the correlation analysis between the differentially expressed lncRNAs and mRNAs In the network, yellow node represents the lncRNAs, and green node represents the target mRNAs. The size of node is proportional to the outgoing link number. The red outgoing link represents up-regulation, the blue outgoing link represents down-regulation.

To determine which lncRNAs and its corresponding mRNAs play critical roles in AMI progression, we constructed a correlated network of the differentially expressed lncRNAs and mRNAs. Correlations between lncRNAs and their corresponding genes (mRNAs) were performed with Pearson's correlation and coefficients obtained with no less than 0.99 were used to construct the network. As shown in Figures 9, 3 source lncRNAs (p2355, p0898_imsncRNA716 and p0734_imsncRNA511 indicated by yellow cycles) were identified that were correlated with different expression (down-regulation or up-regulation) of corresponding genes indicated by green cycles. The size of cycles represented significant correlation, the red line represented positive correlation, and the blue line represented negative correlation. The details of the source lncRNA and their corresponding genes are presented in Table 4. According to the correlated references and other studies, there were 8 corresponding genes related to inflammation, such as CD4, ITGB2, GSR, lnc-ZNHIT2-1, LOC100505564, lnc-ZNHIT2-1, SNRPB and MPG. And there were 10 corresponding genes related to apoptosis, such as CD4, ITGB2, GSR, lnc-ZNHIT2-1, LOC100505564, GLTP, GIT1, TNK2, TYK2 and MPG.

**Table 4:** LncRNA-mRNA network analysis

LncRNA probe	Probe of target gene	Name of target gene	Correlation	*P* value
**p2355**	A_33_P3219517	CD4	0.999	5.33E-12
	A_23_P89727	ITGB2	0.995	2.48E-09
	A_33_P3276784	CTDP1	0.994	5.52E-09
	A_33_P3332744	ARPC4	0.994	5.64E-09
	A_33_P3360684	ERGIC3	0.992	1.41E-08
	A_33_P3387956	JADE2	0.992	1.44E-08
	A_33_P3410011	MAN1C1	0.992	1.74E-08
	A_24_P216313	ATP1A4	0.992	2.06E-08
	A_33_P3233070	GSR	0.991	2.32E-08
	A_24_P226278	lnc-ZNHIT2-1	0.991	2.36E-08
	A_21_P0000664	LOC100505564	0.991	2.73E-08
	A_24_P237778	GLTP	0.991	2.79E-08
	A_32_P31618	---	0.991	2.88E-08
	A_33_P3835524	CYTH1	0.991	2.97E-08
	A_24_P307626	CORO1B	0.991	3.00E-08
	A_33_P3401673	DPP7	0.991	3.23E-08
	A_33_P3266419	GIT1	0.991	3.43E-08
	A_23_P430411	PBXIP1	0.990	3.67E-08
	A_24_P20383	POU2F2	0.990	3.88E-08
**p0734_imsncRNA511**	A_23_P89727	CTDP1	0.998	1.03E-10
	A_33_P3219517	lnc-ZNHIT2-1	0.993	9.34E-09
	p2355	---	0.991	2.86E-08
	A_33_P3276784	---	0.990	4.11E-08
	A_23_P154675	SNRPB	0.995	2.83E-09
	A_23_P147439	ATXN2L	0.992	2.05E-08
	A_33_P3219517	lnc-ZNHIT2-1	0.993	9.34E-09
	A_32_P180741	TNK2	0.995	3.33E-09
	A_23_P165078	ABHD17A	0.991	3.41E-08
	A_24_P289726	PSMD3	0.992	2.15E-08
	A_33_P3382303	FMNL1	0.992	2.42E-08
**p0898_imsncRNA716**	A_33_P3266419	GIT1	–0.994	6.23E-09
	A_33_P3387956	PBXIP1	–0.995	3.14E-09
	p2355	POU2F2	–0.995	3.02E-09
	A_21_P0000664	CD4	–0.995	2.99E-09
	A_23_P141917	TYK2	–0.992	2.24E-08
	A_23_P119377	CYTH2	–0.992	1.42E-08
	A_24_P48162	MPG	–0.990	3.87E-08
	A_23_P59179	RXRB	–0.991	3.12E-08
	A_24_P216313	ERGIC3	–0.991	3.50E-08
	A_24_P20383	ARPC4	–0.993	9.50E-09
	A_33_P3401673	GIT1	–0.990	3.97E-08
	A_24_P200942	TSC22D4	–0.990	3.87E-08
	A_33_P3219517	lnc-ZNHIT2-1	–0.990	3.57E-08
	A_24_P206343	MYO1G	–0.992	1.92E-08
	A_33_P3835524	POU2F2	–0.992	1.94E-08
	A_33_P3410011	PBXIP1	–0.992	1.45E-08
	A_21_P0000664	CD4	–0.995	2.99E-09
	A_32_P31618	GSR	–0.992	2.08E-08

### qRT-PCR validation

**Figure 10:**
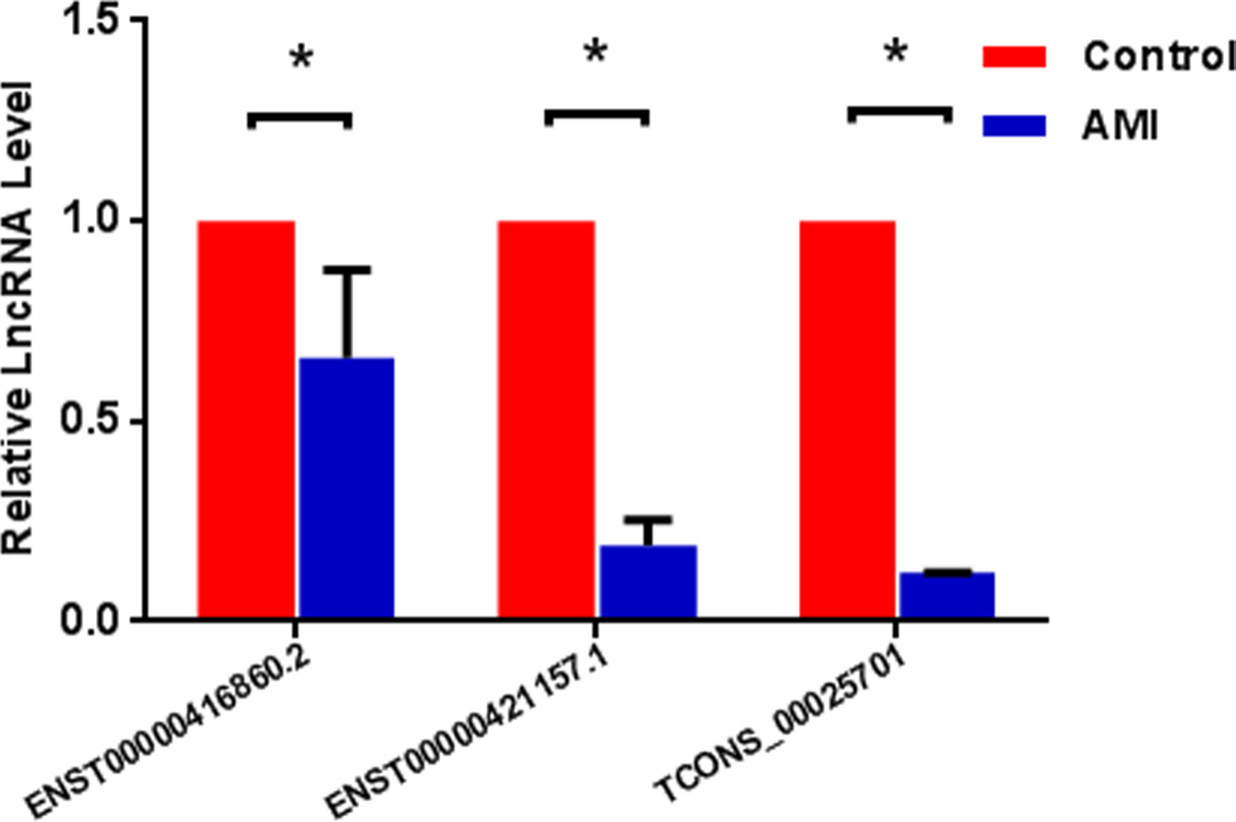
qRT-PCR validation of 3 randomly selected lncRNAs in study participants Compared to healthy controls, expression (log2 fold change) of 3 lncRNAs, ENST00000416860.2, ENST00000421157.1 and TCONS_00025701 was significantly lower in patients with acute myocardial infarction (AMI), results were consistent with the findings obtained from the microarray chip analysis. *P* < 0.05 vs. controls.

To validate the microarray data of lncRNA expression, we assessed the expression level of three randomly selected lncRNAs using qRT-PCR from peripheral blood samples of Uyghur AMI patients (*n* = 50) and healthy controls (*n* = 50). A description of the three down-regulated lncRNAs are presented in Table 5, and the PCR primers of these three lncRNAs are presented in Table 6. As shown in Figure 10, differences in the expression of three lncRNAs were detected in Uyghur AMI patients compared with healthy controls: lncRNA TCONS_00025701 was the most significantly decreased (8.16-fold), followed by lncRNA ENST00000421157.1 (5.29-fold), and lncRNA ENST00000416860.2 (1.52-fold), respectively. These results were consistent with the findings obtained from the microarray chip analysis.

**Table 5:** The randomly selected lncRNAs

lncRNA ID	*P* value	Fold change	Regulation	Probe	Chromosome	Start	End	Class
**ENST00000416860.2**	0.013	2.69	down	p36	1	2481358	2488450	Antisense
**ENST00000421157.1**	0.005	3.05	down	p351	1	113554308	113615727	Divergent
**TCONS_00025701**	0.030	5.80	down	p20189	17	55122437	55128557	Intergenic

**Table 6:** The PCR primers of the randomly selected lncRNAs

Gene name	Forward primer	Reverse primer	Hybridization temperature (°c)
**ENST00000416860.2**	TTTTCGGAGGCAGGTTCCAG	CACCTCGCATGTGCGTTTAT	60
**ENST00000421157.1**	TGGCACTGGGCACTTGATAA	CAAGTGTGCAGTAGGTATTAGCC	60
**TCONS_00025701**	GAGGAGCAGTTCATCCCAGTC	GCAACCACCTGTTCCACGA	60

## DISCUSSION

AMI, the most common heart diseases, is a major cause of death and disability worldwide and exerts huge burdens on the society [16]. Identification of novel biological information or biomarkers which may have a potential in better stratification and management of patients with acute coronary artery syndrome. LncRNAs had long been considered as simply transcriptional noise [17]. However, recent studies have showed that lncRNAs play important roles in pathophysiology of cardiovascular diseases involving in regulations of basal transcription, post-transcriptional processes, DNA methylation, epigenetic modifications, histone modification, directly bind proteins and protein function [18–21]. LncRNAs are now emerging as a novel class of gene regulators to cardiovascular diseases [22].

Following the exploration in expression pattern of lncRNAs in some types of cardiovascular disease including AMI in other populations, we investigated the genome-wide expression profile of lncRNAs in Uyghur ethnicity in China. Using microarray, expression profiles of lncRNA and mRNA in 5 Uyghur AMI patients and 5 Uyghur healthy volunteers were studied. A total 3624 up- and 1637 down-regulated lncRNA probes were identified to be significantly and differentially expressed in AMI patients and accordingly in mRNA levels, 1833 mRNAs were up-regulated and 2063 mRNAs were down-regulated when compared with that in healthy controls. There are several studies investigated the relationship between lncRNAs and AMI. Ounzain S *et al*. found that there were 988 (67 up-regulated and 66 down-regulated) UCSC-annotated lncRNAs and 1521 (86 up-regulated and 225 down-regulated) novel unannotated lncRNAs in AMI patients [12]. Liu Y *et al*. studied expression profile and ontology analysis of lncRNAs in post-ischemic heart, they found that 64 lncRNAs were up-regulated and 87 down-regulated, accordingly, they also revealed 50 mRNAs were up-regulated and 60 down-regulated in the ischemia-reperfused murine myocardium [23].

Further, in our study, the potential function of lncRNAs and their co-expressed mRNAs were predicted. The most enriched pathways corresponding to the dysregulation of lncRNAs in related to AMI were apoptosis and its corresponding p53 signaling pathway. Other dysregulated lncRNAs were related to inflammatory signaling pathway and contraction of cardiac muscle and vascular smooth muscle. These information suggest that there might be coordinated patterns between lncRNAs and its co-expressed mRNAs involved in the development of AMI. Furthermore, we analyzed the relation between altered lncRNAs and the network of inflammation- and apoptosis-related mRNAs. We found that the lncRNAs (p2355, p0898_imsncRNA716, p0734_imsncRNA511) and their co-expressed mRNAs, such as CD4, ITGB2, GSR, lnc-ZNHIT2-1, LOC100505564, lnc-ZNHIT2-1, SNRPB and MPG were related to inflammation. These lncRNAs and their co-expressed mRNAs, such as CD4, ITGB2, GSR, lnc-ZNHIT2-1, LOC100505564, GLTP, GIT1, TNK2, TYK2 and MPG, related to the apoptosis. We speculated that these lncRNAs and their co-expressed mRNAs may participate in AMI occurrence and development. However, this hypothesis needs to be further investigated.

Some lncRNAs have been reported to be potential biomarkers in cardiovascular disease. The mitochondrial lncRNA uc022bqs.1 (LIPCAR) was downregulated early after AMI but upregulated during later stages which identified patients developing cardiac remodeling and were independently to other risk markers associated with future cardiovascular deaths [24]. Increased expression of MIAT [25] and circulating lncRNA OTTHUMT00000387022 in monocytes [26] have been reported to link with a high risk of CAD. LncRNA, ANRIL expression is associated with the chromosome 9p21 genotype and correlated with atherosclerosis severity [27] and further ANRIL may also increase the risk of ischemic stroke through regulation of the CARD8 pathway [28]. The lncRNA MIAT has been reported as a biomarkers for severe LV remodeling after AMI [29], and the circulating levels of lncRNAs, such as ANRIL [30] and lncRNA-P21 [31] were also increased in atherosclerosis which may be important in its pathogenesis. Compared to these reports, we found in our study that lncRNA, MIAT, was decreased in Uyghur AMI patients versus healthy controls (FC value was 3.11, *P* = 0.006), and other lncRNAs such as ANRIL, LIPCAR, lncRNA-P21 and OTTHUMT00000387022 were not detected. However, we found that the expression levels of lncRNA ENST00000416860.2, ENST00000421157.1 and TCONS_00025701 were decreased in AMI patients, which identified previously non-reported novel lncRNAs with altered expression in the setting of AMI. Our results provide a supplementary data in this field.

We have to admit the limitation of this study, as the participants in this study were from a single center and only tested in one ethnic group, whether there is a difference in patients from different areas and races that is unknown. Therefore, its validity should be tested further in a large population.

In conclusion, the present study using microarray approach to examine the expression profile of lncRNA in peripheral blood from Uyghur AMI patients in comparison with matched healthy controls. Results provide previously unreported bioinformation on genomic-wide lncRNA expression and corresponding mRNA expression in Uyghur AMI patients. Potential functional link in post-MI revealed dysregulated lncRNA expression involving a variety of biological and pathological processes. Notably, top 20 aberrantly expressed lncRNA were related to various aspects of inflammatory regulation and apoptosis. These findings provide useful bioinformation with functional links, which may have a potential role in the development of AMI.

## MATERIALS AND METHODS

### Patients and sample collection

This study was approved by the Ethics Committee of the First Affiliated Hospital of Xinjiang Medical University and was conducted according to the standards of the Declaration of Helsinki. Written informed consent was obtained from all the participants. Patients with AMI treated with primary percutaneous coronary intervention were recruited in this study. AMI patients was defined by the following: (a) occluded major coronary artery: TIMI (thrombolysis in MI), 0 flow in the left anterior descending, circumflex, or right coronary artery; (b) peak creatine kinase (CK) activity >600 U/L (3 times higher than the upper limit of the reference interval). (c) positive troponin T (TnT) concentration >0.15 μg/L. Exclusion criteria included the following: (a) acute coronary syndrome, heart failure and severe valvular heart disease, (b) infectious processes in last 4 weeks or active chronic inflammatory disease, (c) other severe systemic diseases (such as renal failure and hepatic disease), malignant tumor, and autoimmune diseases or patients who took anti-inflammatory drugs, (d) other inflammatory diseases. Blood samples were collected between December 2014 and September 2015 at the First Affiliated Hospital of Xinjiang Medical University. Five AMI patients and 5 healthy volunteers were randomly selected using coding system (AMI-U1, AMI-U2, AMI-U3, AMI-U6, AMI-U8) and (Normal N-U2, N-U3, N-U4, N-U5, N-U6) for lncRNA chip analysis. In addition, blood samples from the rest of 50 AMI patients and 50 healthy volunteers were obtained to validate the lncRNA expression level using qRT-PCR.

### RNA extraction, labeling and hybridization

Total RNA was extracted from peripheral blood cells using the Trizol reagent (Invitrogen) and purified with mirVana miRNA Isolation Kit (Ambion, Austin, TX, USA) according to manufacter's protocol. The purity and concentration of RNA were determined from OD260/280 readings using spectrophotometer (NanoDrop ND-1000). RNA integrity was determined by 1% formaldehyde denaturing gel electrophoresis. Sample labeling and array hybridization were performed according to the Agilent One-Color Microarray-Based Gene Expression Analysis protocol (Agilent Technology). cDNA labeled with a fluorescent dye (Cy5 and Cy3-dCTP) was produced by Eberwine's linear RNA amplification method and subsequent enzymatic reaction [32]. Double-stranded cDNAs (containing the T7 RNA polymerase promoter sequence) were synthesized from 1ug total RNA using the CbcScript reverse transcriptase with cDNA synthesis system according to the manufacturer's protocol (Capitalbio) with the T7 Oligo (dT) and T7 Oligo (dN) [33]. After completion of double-stranded cDNA (dsDNA) synthesis using DNA polymerase and RNase H, the dsDNA products were purified using a PCR NucleoSpin Extract II Kit (MN) and eluted with 30 μL elution buffer. The eluted double-stranded cDNA products were vacuum evaporated to 16 μL and subjected to 40 μL *in vitro* transcription reactions at 37°C for 14 h using a T7 Enzyme Mix. The amplified cRNA was purified using the RNA Clean-up Kit (MN).

Klenow enzyme labeling strategy was adopted after reverse transcription using CbcScript II reverse transcriptase [34]. Briefly, 2 μg amplified RNA was mixed with 4 μg random nanomer, denatured at 65°C for 5 min, and cooled on ice. Then, 5 μL of 4×first-strand buffer, 2 μL of 0.1M DTT, and 1.5 μL CbcScript II reverse transcriptase were added. The mixtures were incubated at 25°C for 10 min, then at 37°C for 90 min. The cDNA products were purified using a PCR NucleoSpin Extract II Kit (MN) and vacuum evaporated to 14 μL. The cDNA was mixed with 4 μg random nanomer, heated to 95°C for 3 min, and snap cooled on ice for 5 min. Then, 5 μL Klenow buffer, dNTP, and Cy5-dCTP or Cy3-dCTP (GE Healthcare) were added to final concentrations of 240 μM dATP, 240 μM dGTP, 240 μM dTTP, 120 μM dCTP, and 40 μM Cy-dCTP. Klenow enzyme (1.2 μL) was then added, and the reaction was performed at 37°C for 90 min. Labeled cDNA was purified with a PCR NucleoSpin Extract II Kit (MN) and resuspended in elution buffer.

Labeled controls and test samples labeled with Cy5-dCTP and Cy3-dCTP were dissolved in 80 μL hybridization solution containing 3×SSC, 0.2% SDS, 5×Denhardt's solution and 25% formamide. DNA in hybridization solution was denatured at 95°C for 3 min prior to loading onto a microarray. Arrays were hybridized in a Agilent Hybridization Oven overnight at a rotation speed of 20 rpm and a temperature of 42°C, and then washed with two consecutive solutions (0.2% SDS, 2× SSC at 42°C for 5 min, and 0.2× SSC for 5 min at room temperature).

### LncRNA and mRNA microarrays

Approximately 200 ng of total RNA from each sample was used for the lncRNA microarray analysis. LncRNA expression was analyzed using Agilent human lncRNA + mRNA Array V4.0 (4 × 180 K format), with each array containing probes interrogating about 41,000 human lncRNAs and about 34,000 human mRNAs (Agilent, USA). Those lncRNA and mRNA target sequences were merged from multiple databases, 23898 from GENCODE/ENSEMBL, 21488 from LNCipedia, 14353 from Human LincRNA Catalog [35], 13701 from NRED (ncRNA Expression Database), 7760 from RefSeq, 5627 from USCS, 3019 from LncRNAs-a (Enhancer-like), 1053 from Antisense ncRNA pipeline, 1038 from H-InvDB, 962 UCRs, 848 from Chen Ruisheng lab (Institute of Biophysics, Chinese Academy of Science) and 407 Hox ncRNAs. Each RNA was detected by probes that were replicated 2 times. The array also contained 4974 control probes (Agilent, USA).

### Microarray imaging and data analysis

The lncRNA and mRNA array data were analyzed for data summarization, normalization and quality control with use of GeneSpring software V13.0 (Agilent). Differentially expressed lncRNAs with statistical significance were identified through Volcano Plot filtering and hierarchical clustering. Differentially expressed genes were identified through the random variance model and *p* values were calculated using the paired *t*-test. The significance thresholds established for the up- and down-regulated genes required a fold change (FC) ≥ 2.0 and a *P* ≤ 0.05. The data was Log 2 transformed and median centered by genes using the Adjust Data function of CLUSTER 3.0 software. Data were then further analyzed using hierarchical clustering with average linkage. Finally, tree visualization was performed with use of Java Treeview (Stanford University School of Medicine, Stanford, CA, USA).

### LncRNA related functional and pathway analyses

GO analysis is a functional analysis for relating differentially expressed mRNAs with GO categories. The GO categories were derived from the Gene Ontology Consortium (www.geneontology.org), which describes gene product attributes within three defined terms: biological processes, molecular functions and cellular components. The *P*-value denotes the significance of the GO term enrichment. The lower the *P*-value, the more significant the GO term (a *P* < 0.05 is recommended).

Pathway analysis is a functional analysis that maps genes to the Kyoto Encyclopedia of Genes and Genome (KEGG) pathways. It was used to determine the main pathway of the differentially expressed genes according to KEGG. Fisher's exact test and χ^2^ tests were used to select the main pathway, and the significance threshold was defined by *p* value and false discovery rate (FDR) [36]. The lower the *p*-value, the more significant the pathway (the *P*-value cut-off is 0.05).

### Construction of the coding-non-coding gene co-expression network

The coding-non-coding gene co-expression network (CNC network) was constructed based on correlation analysis between the differential expressed lncRNAs and mRNAs. For each pair of genes, a Pearson correlation coefficient was calculated and the significant correlation pairs were selected for construction of the network. LncRNAs and mRNAs with Pearson correlation coefficients not less than 0.99 were selected to draw the network though the open source bioinformatics software Cytoscape. In a network analysis, a degree centrality is defined as the link numbers between nodes. A degree is the simplest and most important measure of a gene centrality within a network that determines its relative importance [37].

### qRT-PCR

Quantitative real-time polymerase chain reaction (qRT-PCR) was used to validate the lncRNA expression level. In brief, the total RNA was isolated using Trizol reagent (Invitrogen) and then reverse transcribed to cDNA using RNeasy Mini Kit (QIAGEN, China) in accordance to the manufacturer's protocol. Primers of each lncRNA were designed using Primer 5.0 and checked with the Basic Local Alignment Search Tool (BLAST) from NCBI to confirm that the amplified product was unique. The primer sequences used are shown in Table 6. Real-time PCR was performed using Power SYBR Green PCR Master (Applied Biosystems, USA) in 7900 HT Fast RealTime PCR system (Applied Biosystems, USA). The threshold cycle value (Ct) of each product was determined and Expression levels were calculated using the method of 2^−ΔΔct^ and normalized to the internal control of GAPDH. The level of each lncRNA in MI patients was expressed as the fold changes against the averaged level of the same lncRNA in healthy controls.

### Statistical analysis

The data were analyzed with IBM SPSS Statistics 18.0 software (SPSS Inc, Chicago, ILLinois, USA). Differential expression levels of lncRNAs were compared via independent-sample *t*-tests between two groups. GO and pathway analyses were evaluated using Fisher's exact test. All values are expressed as the mean ± standard deviation, and significance was assigned at the *P < 0.05* level.

## References

[1] Naghavi M, Libby P, Falk E, Casscells SW, Litovsky S, Rumberger J, Badimon JJ, Stefanadis C, Moreno P, Pasterkamp G, Fayad Z, Stone PH, Waxman S (2003). From vulnerable plaque to vulnerable patient: a call for new definitions and risk assessment strategies: Part II. Circulation.

[2] McPherson R (2010). Chromosome 9p21 and coronary artery disease. N Engl J Med.

[3] Ardissino D, Berzuini C, Merlini PA, Mannuccio MP, Surti A, Burtt N, Voight B, Tubaro M, Peyvandi F, Spreafico M, Celli P, Lina D, Notarangelo MF (2011). Influence of 9p21.3 genetic variants on clinical and angiographic outcomes in early-onset myocardial infarction. J Am Coll Cardiol.

[4] Peters T, Schroen B (2014). Missing links in cardiology: long non-coding RNAs enter the arena. Pflugers Arch.

[5] Ponting CP, Belgard TG (2010). Transcribed dark matter: meaning or myth?. Hum Mol Genet.

[6] Prensner JR, Chinnaiyan AM (2011). The emergence of lncRNAs in cancer biology. Cancer Discov.

[7] Grote P, Wittler L, Hendrix D, Koch F, Wahrisch S, Beisaw A, Macura K, Blass G, Kellis M, Werber M, Herrmann BG (2013). The tissue-specific lncRNA Fendrr is an essential regulator of heart and body wall development in the mouse. Dev Cell.

[8] Klattenhoff CA, Scheuermann JC, Surface LE, Bradley RK, Fields PA, Steinhauser ML, Ding H, Butty VL, Torrey L, Haas S, Abo R, Tabebordbar M, Lee RT (2013). Braveheart, a long noncoding RNA required for cardiovascular lineage commitment. Cell.

[9] Yang KC, Yamada KA, Patel AY, Topkara VK, George I, Cheema FH, Ewald GA, Mann DL, Nerbonne JM (2014). Deep RNA sequencing reveals dynamic regulation of myocardial noncoding RNAs in failing human heart and remodeling with mechanical circulatory support. Circulation.

[10] Wang K, Liu F, Zhou LY, Long B, Yuan SM, Wang Y, Liu CY, Sun T, Zhang XJ, Li PF (2014). The long noncoding RNA CHRF regulates cardiac hypertrophy by targeting miR-489. Circ Res.

[11] Gupta SK, Piccoli MT, Thum T (2014). Non-coding RNAs in cardiovascular ageing. Ageing Res Rev.

[12] Ounzain S, Micheletti R, Beckmann T, Schroen B, Alexanian M, Pezzuto I, Crippa S, Nemir M, Sarre A, Johnson R, Dauvillier J, Burdet F, Ibberson M (2015). Genome-wide profiling of the cardiac transcriptome after myocardial infarction identifies novel heart-specific long non-coding RNAs. Eur Heart J.

[13] Yan YZ, Ma RL, Ding YS, Guo H, Zhang JY, Mu LT, Zhang M, Liu JM, Rui DS He J, Sun F, Wang K, Guo SX (2015). Association of Inflammation with Metabolic Syndrome among Low-Income Rural Kazakh and Uyghur Adults in Far Western China. Mediators Inflamm.

[14] Clark MB, Johnston RL, Inostroza-Ponta M, Fox AH, Fortini E, Moscato P, Dinger ME, Mattick JS (2012). Genome-wide analysis of long noncoding RNA stability. Genome Res.

[15] Wang H, Zheng H, Azuaje F (2007). Poisson-based self-organizing feature maps and hierarchical clustering for serial analysis of gene expression data. IEEE/ACM Trans Comput Biol Bioinform.

[16] Chwojnicki K, Wierucki L, Zagozdzon P, Wojtyniak B, Nyka WM, Zdrojewski T (2016). Long-term mortality after stroke is higher than after myocardial infarction. Neurol Sci.

[17] Gibb EA, Vucic EA, Enfield KS, Stewart GL, Lonergan KM, Kennett JY, Becker-Santos DD, MacAulay CE, Lam S, Brown CJ, Lam WL (2011). Human cancer long non-coding RNA transcriptomes. Plos One.

[18] Yoon JH, Abdelmohsen K, Gorospe M (2013). Posttranscriptional gene regulation by long noncoding RNA. J Mol Biol.

[19] Arun G, Akhade VS, Donakonda S, Rao MR (2012). mrhl RNA, a long noncoding RNA, negatively regulates Wnt signaling through its protein partner Ddx5/p68 in mouse spermatogonial cells. Mol Cell Biol.

[20] Mohammad F, Pandey GK, Mondal T, Enroth S, Redrup L, Gyllensten U, Kanduri C (2012). Long noncoding RNA-mediated maintenance of DNA methylation and transcriptional gene silencing. Development.

[21] Chu C, Qu K, Zhong FL, Artandi SE, Chang HY (2011). Genomic maps of long noncoding RNA occupancy reveal principles of RNA-chromatin interactions. Mol Cell.

[22] Vausort M, Wagner DR, Devaux Y (2014). Long noncoding RNAs in patients with acute myocardial infarction. Circ Res.

[23] Liu Y, Li G, Lu H, Li W, Li X, Liu H, Li X, Li T, Yu B (2014). Expression profiling and ontology analysis of long noncoding RNAs in post-ischemic heart and their implied roles in ischemia/reperfusion injury. Gene.

[24] Kumarswamy R, Bauters C, Volkmann I, Maury F, Fetisch J, Holzmann A, Lemesle G, de Groote P, Pinet F, Thum T (2014). Circulating long noncoding RNA, LIPCAR, predicts survival in patients with heart failure. Circ Res.

[25] Ishii N, Ozaki K, Sato H, Mizuno H, Saito S, Takahashi A, Miyamoto Y, Ikegawa S, Kamatani N, Hori M, Saito S, Nakamura Y, Tanaka T (2006). Identification of a novel non-coding RNA, MIAT, that confers risk of myocardial infarction. J Hum Genet.

[26] Cai Y, Yang Y, Chen X, Wu G, Zhang X, Liu Y, Yu J, Wang X, Fu J, Li C, Jose PA, Zeng C, Zhou L (2016). Circulating ‘lncRNA OTTHUMT00000387022′ from monocytes as a novel biomarker for coronary artery disease. Cardiovasc Res.

[27] Holdt LM, Hoffmann S, Sass K, Langenberger D, Scholz M, Krohn K, Finstermeier K, Stahringer A, Wilfert W, Beutner F, Gielen S, Schuler G, Gabel G (2013). Alu elements in ANRIL non-coding RNA at chromosome 9p21 modulate atherogenic cell functions through trans-regulation of gene networks. Plos Genet.

[28] Bai Y, Nie S, Jiang G, Zhou Y, Zhou M, Zhao Y, Li S, Wang F, Lv Q, Huang Y, Yang Q, Li Q, Li Y (2014). Regulation of CARD8 expression by ANRIL and association of CARD8 single nucleotide polymorphism rs2043211 (p.C10X) with ischemic stroke. Stroke.

[29] de Gonzalo-Calvo D, Kenneweg F, Bang C, Toro R, van der Meer RW, Rijzewijk LJ, Smit JW, Lamb HJ, Llorente-Cortes V, Thum T (2016). Circulating long-non coding RNAs as biomarkers of left ventricular diastolic function and remodelling in patients with well-controlled type 2 diabetes. Sci Rep.

[30] Kumarswamy R, Bauters C, Volkmann I, Maury F, Fetisch J, Holzmann A, Lemesle G, de Groote P, Pinet F, Thum T (2014). Circulating Long Noncoding RNA, LIPCAR, Predicts Survival in Patients with Heart Failure. Circ Res.

[31] Bai Y, Nie S, Jiang G, Zhou Y, Zhou M, Zhao Y, Li S, Wang F, Lv Q, Huang Y, Yang Q, Li Q, Li Y (2014). Regulation of CARD8 Expression by ANRIL, Association of CARD8 Single Nucleotide Polymorphism Rs2043211 (P.C10X) with Ischemic Stroke. Stroke.

[32] Sun J, Wu J (2015). Expression profiling of long noncoding RNAs in neonatal and adult mouse tests. Data Brief.

[33] Gao W, Hillwig ML, Huang L, Cui G, Wang X, Kong J, Yang B, Peters RJ (2009). A functional genomics approach to tanshinone biosynthesis provides stereochemical insights. Org Lett.

[34] Li H, Li H, Yue H, Wang W, Yu L, ShuoWang Cao Y, Zhao J (2016). Comparison between smaller ruptured intracranial aneurysm and larger un-ruptured intracranial aneurysm: gene expression profile analysis. Neurosurg Rev.

[35] Orom UA, Derrien T, Beringer M, Gumireddy K, Gardini A, Bussotti G, Lai F, Zytnicki M, Notredame C, Huang Q, Guigo R, Shiekhattar R (2010). Long noncoding RNAs with enhancer-like function in human cells. Cell.

[36] Kanehisa M, Goto S, Kawashima S, Okuno Y, Hattori M (2004). The KEGG resource for deciphering the genome. Nucleic Acids Res.

[37] Barabasi AL, Oltvai ZN (2004). Network biology: understanding the cell’s functional organization. Nat Rev Genet.

